# Diabetic Myonecrosis: An Atypical Presentation

**DOI:** 10.1155/2013/190962

**Published:** 2013-06-03

**Authors:** José Hernán Martínez, Oberto Torres, Michelle M. Mangual García, Coromoto Palermo, María de Lourdes Miranda, Eva González, Ignacio Chinea Espinoza, Ivan Laboy, Mirelis Miranda, Kyrmarie Dávila, Rafael Tirado, Mildred Padilla

**Affiliations:** ^1^Department of Endocrinology, 4th Floor, San Juan City Hospital, CMMS No. 79, P.O. Box 70344, San Juan, PR 00936-8344, USA; ^2^Internal Medicine Department, San Juan City Hospital, CMMS No. 79, P.O. Box 70344, San Juan, PR 00936-8344, USA

## Abstract

Diabetic myonecrosis is a frequently unrecognized complication of longstanding and poorly controlled diabetes mellitus. The clinical presentation is swelling, pain, and tenderness of the involved muscle, most commonly the thigh muscles. Management consists of conservative measures including analgesia and rest. Short-term prognosis is good, but long-term prognosis is poor with most patients dying within 5 years. Failure to properly identify this condition will expose the patient to aggressive measures that could result in increased morbidity. To our knowledge this is the first case reported in which there was involvement of multiple muscle groups including upper and lower limbs.

## 1. Introduction

Diabetic muscle infarction (DMI), also named diabetic myonecrosis, is a rare and commonly underdiagnosed complication of longstanding diabetes mellitus. It refers to spontaneous ischemic necrosis of skeletal muscles not related to atheroembolism or occlusion of major arteries and is one of many micro- and macrovascular complications of diabetes. The usual presentation is sudden onset of pain at the involved muscles associated with swelling and tenderness [1]. The thigh muscles (usually the vastus group) are most commonly affected, but calf muscles might be involved as well. One case of upper limb involvement is also reported. Bilateral involvement has been described in 8.4% of cases [2]. We report a case of diabetic myonecrosis involving bilateral upper extremities that progressed to involve the lower extremities. 

## 2. Case Report

This is a case of a 49-year-old male patient, with 12 years history of poorly controlled diabetes mellitus type 2, on insulin therapy. Diabetic neuropathy, nephropathy, previous right toe amputation due to peripheral arterial disease, hypertension, and dyslipidemia were clinically evident. He arrived to our urgency room with the chief complaint of bilateral proximal upper extremities and upper back pain, weakness and stiffness of three days of evolution. Pain was initially of moderate intensity and during the course of one week became excruciating in nature associated with shoulder movement restriction and swelling. Patient was known to be a noncompliant type 2 diabetic on insulin therapy with no history of direct or indirect trauma, abnormal exercise, arthralgia, fever, nausea, vomiting, or skin breakdown, denied injecting insulin in his thighs or arms, use of tobacco or illicit drugs, but admitted a history of excessive alcohol consumption for a period of 2 years. Currently he is unemployed due to diabetes mellitus complications but was formerly an electrician. His family history was remarkable for his mother, father, and one only sibling with diabetes mellitus type 2.

**Figure 1:**
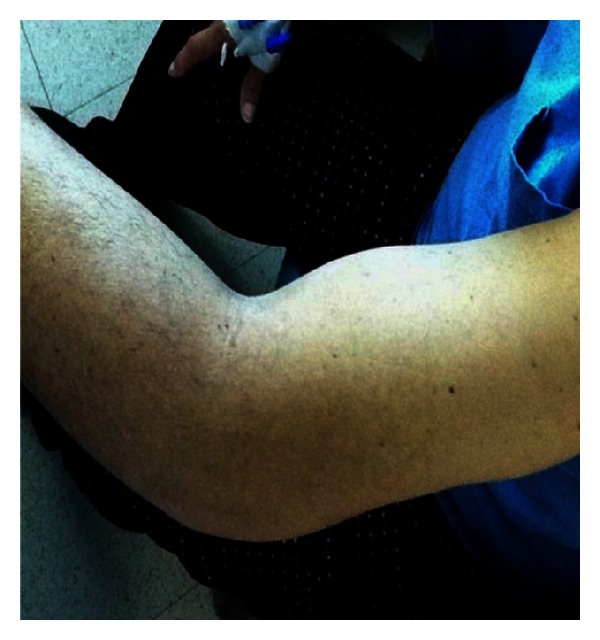
Left arm nonpitting edema with induration.

**Figure 2:**
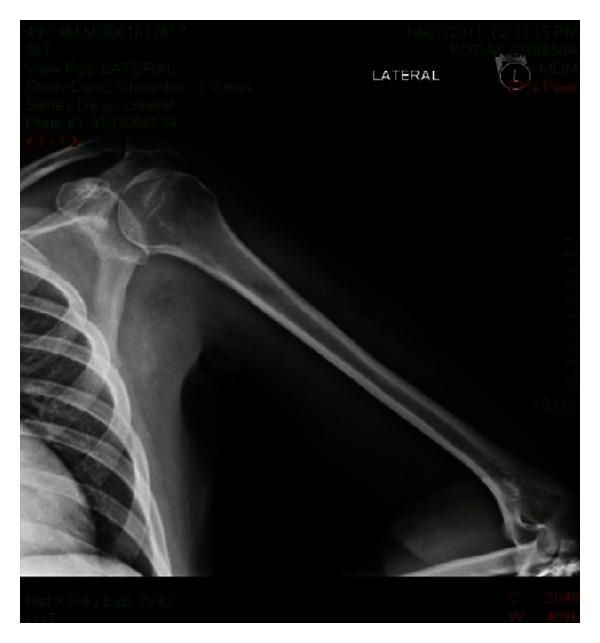
Left shoulder AP X-ray. No subcutaneous gas. Soft tissue swelling.

**Figure 3:**
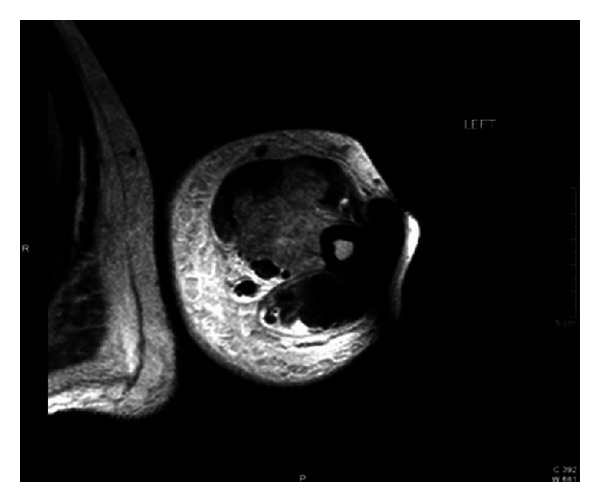
Left upper extremity MRI. T2 hyperintensity of the deep biceps, brachialis, and brachioradialis muscles in the anterior compartment.

**Figure 4:**
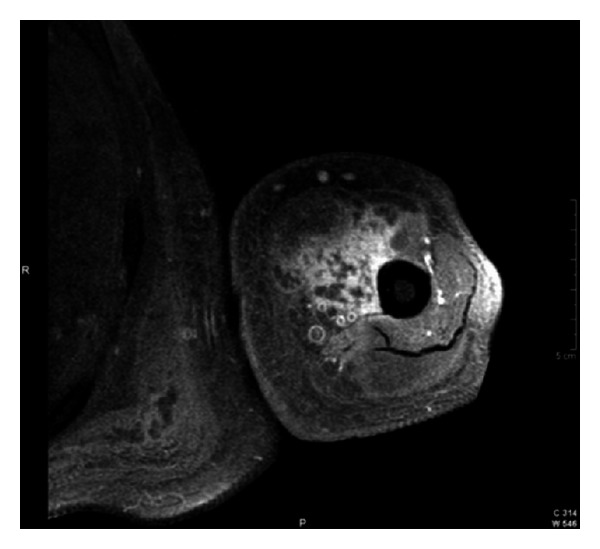
Left upper extremity MRI. T1 weighted image. Heterogenous patchy contrast enhancement consistent with myonecrosis.

**Figure 5:**
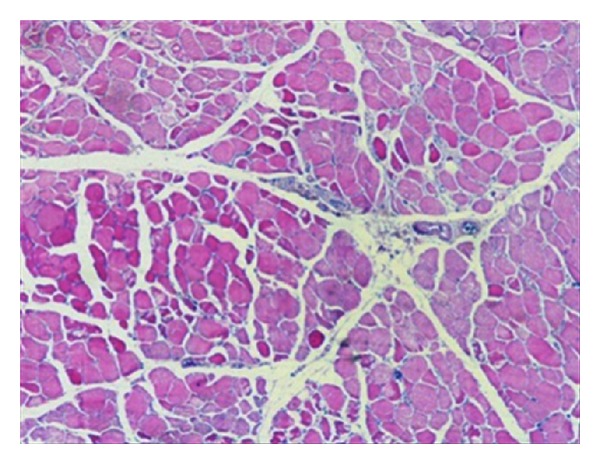
Muscle biopsy light microscopy. There is no evidence of necrosis, atrophy, fibrosis, abnormal accumulations, nor vasculitis.

**Figure 6:**
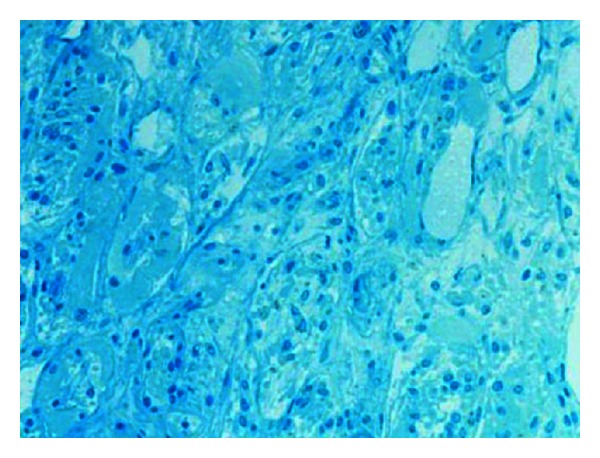
Muscle biopsy immunofluorescence. Negative endo- or perimysial IgG, IgA, IgM, and C3.

On physical examination patient was active, alert, and oriented in time, person, and place. Vital signs are blood pressure 98/56 mmHg, pulse rate 94 bpm, and respiratory rate 20 bpm. He was afebrile with a temperature of 36.4°C and adequate oxygen saturation (97%) at room air. His body mass index was 24.6 kg/m^2^. Fundoscopic evaluation revealed moderate bilateral preproliferative diabetic retinopathy. Neck was supple without carotid bruits. Cardiac examination showed mild tachycardia with a regular rhythm and no murmurs, rubs, or gallops. The point of maximal impulse was not displaced. Lungs were clear to auscultation. Abdomen was nontender, nondistended, with bowel movements present and normoactive. Bilateral upper extremities showed nonpitting edema with local tenderness and induration around the proximal region, without palpable crepitus or erythema (Figure 1). Peripheral pulses were present and symmetrical. Patient's neurologic evaluation revealed decreased sensation to 10 g monofilament testing noted at the plantar side of each foot. Laboratory tests reported a random blood glucose of 292 mg/dL, leukocyte count of 7.1 × 10^3^ cells/L, and a hemoglobin concentration of 12 g/dL with a mean corpuscular volume of 86 fl. Chemistry showed an elevated creatinine of 1.9 mg/dL, serum potassium of 6.9 mEq/L, CO_2_ of 19.9, and a serum creatine kinase of 225 U/L (normal value 60–110 U/L). Other electrolytes were within normal limits. Glycosylated hemoglobin was elevated at 11.2%, and urinalysis showed proteinuria. Erythrocyte sedimentation rate was 76 mm/hr. Electrocardiogram demonstrated sinus tachycardia with peaked T waves, consistent with previous finding of hyperkalemia, treated immediately with calcium gluconate for cardiac membrane stabilization, insulin for intracellular shifting of potassium and sodium polystyrene sulfonate to decrease potassium absorption. Anteroposterior view roentgenograms of the shoulders were performed without any bone abnormalities or subcutaneous gas but with soft tissue swelling (Figure 2). The patient was admitted and treated initially with one-week course of corticosteroids given that polymyositis was suspected. There was no improvement in muscle pain and weakness, and CK levels were persistently elevated. The ANA test and blood cultures were negative. Corticosteroid therapy was discontinued, and 2 weeks after initial presentation, he started to present the same symptoms on both thighs. At the same time, there was a gradual relief of upper extremities affliction with residual muscle wasting. Upper extremities venous doppler and left arm MRI were performed. Venous Doppler excluded deep venous thrombosis as the cause of the symptoms. The MRI showed T2 hyperintensity of the deep biceps muscle, brachialis, and brachioradialis muscles in the anterior compartment with heterogenous patchy contrast enhancement that with the clinical presentation was consistent with diabetic myonecrosis (Figures 3 and 4). Since this was an atypical presentation of diabetic myonecrosis a muscle biopsy of the left anterior compartment of the arm was performed. Light microscopy showed that the striate muscle was viable and well preserved, with no evidence of necrosis, atrophy, fibrosis, abnormal accumulations, or vasculitis (Figure 5). Immunofluorescence was negative for endo- or perimysial IgG, IgA, IgM, and C_3_ (Figure 6). Although it did not confirm the diagnosis of diabetic muscle infarction, it excluded other etiologies such as inflammatory muscle disease or pyomyositis. Rest, analgesia, and tight glycemic control resolved symptoms over the course of 4 weeks. 

## 3. Discussion

Diabetic muscle infarction, also referred to as diabetic myonecrosis, is an uncommon manifestation of longstanding and poorly controlled diabetes mellitus. The pathogenesis is unknown but may involve hypoxia-reperfusion injury, atherosclerotic occlusion, or vasculitis with thrombosis. Other proposed theories are atheroembolism of small vessels and hypercoagulable state. The most accepted hypothesis at this moment is arteriosclerosis. In a previous study performed by Chester and Banker, they suggested an atheroembolic phenomenon, but no embolic material was found in the cases they reviewed. Instead, the results of the study were more consistent with arteriosclerosis obliterans [3]. Other authors have found evidence consistent with a hypercoagulable state as the etiology of diabetic myonecrosis involving increased factor VII activity, increased levels of plasminogen activator inhibitor and thrombomodulin [4]. The presences of antiphospholipid antibodies in diabetic patients that develop DMI have also been suggested as an etiology of hypercoagulable state responsible for this diabetes complication [5–7]. However, results are not consistent and more research is needed. 

Angervall and Stener first described this condition in 1965 [8]. A recent systematic review of the literature up to August, 2001 identified a total of 116 patients and showed that this complication was more common in women (61.5% of all cases) and in longstanding diabetes (mean 14.3 years). It is frequently unilateral with occasional bilateral involvement with predilection of the thigh muscles, the calf being the second most commonly affected site. Vascular complications of diabetes were present in the majority of cases, particularly nephropathy (71%), retinopathy (59%), and neuropathy (55%) [2]. 

 Clinical manifestations include acute or subacute pain, swelling, and tenderness, typically at the thigh or calf. The first case involving bilateral upper extremities was reported by Mukhopadhyay and colleagues in 2011 [9]. We present a case never seen before in the medical literature, in which involvement of multiple muscle groups including upper and lower limbs was the most remarkable and conspicuous findings.

 Similar presentation may also be seen in pyomyositis, necrotizing fasciitis, deep vein thrombosis, soft tissue abscess, cellulitis, hematoma, and acute compartment syndrome. A recent study indicated that hyper-CK-emia was seen in one-fifth of diabetic patients. A considerable number of these cases were attributable to a primary myopathy, mostly mitochondrial myopathies. For this reason they proposed that an elevated level of creatine kinase in diabetic patients should require further neurologic workup [10]. In this case creatine kinase levels returned to baseline after resolution of symptoms which excluded a primary myopathy as the etiology. Therefore diabetic myonecrosis should be considered in the differential diagnosis in diabetic patients who present with painful swollen muscles. Awareness of the syndrome and the presence of characteristic clinical features suggest the diagnosis. Laboratory and imaging studies aimed at excluding other disorders. The MRI is the imaging test of choice since it is noninvasive, with a better sensitivity and anatomic definition [11]. The gold standard for the diagnosis is muscle biopsy that may be necessary for confirmation when the presentation is atypical as in this case. Needle biopsy is preferred over excisional and incisional biopsies given the potential complications [2]. 

 DMI is a self-limiting disease that resolves spontaneously over a few weeks to months with conservative management including rest and analgesia. Surgical management should be avoided given that patients who undergo surgery have an average recovery period of 13 weeks compared to 5.5 weeks for those who received conservative treatment only as described by Kapur and McKendry [12]. There are other complications associated with surgical intervention such as seroma, hematoma, delayed wound healing, and infection. In the study performed by Kapur and colleagues, medical management including therapy with steroids and antiplatelet agents demonstrated shorter recovery times, but the difference, when compared with the conservative strategy, was not statistically significant, and both groups had similar recurrence rates of 40% with a 2-year mortality of 10% [12]. The short-term prognosis of DMI is good, but the long-term outlook is poor, reflecting the underlying arteriopathy, with death from a major vascular event occurring within 5 years in the majority of patients.

## 4. Conclusion

Diabetic muscle infarction is an uncommon complication of diabetes mellitus that is probably underdiagnosed. It presents clinically with pain, edema, and a palpable mass most commonly in a thigh, but other locations have been reported. Diagnosis is made by means of the clinical presentation and radiologic imaging with the MRI being the most sensitive test. Muscle biopsy is the gold standard but is rarely needed and is recommended only when there is an atypical presentation or symptoms are not improving. It is important to recognize this entity since prompt management with rest, analgesia, and tight glycemic control will improve the symptoms. Avoidance of surgical intervention is crucial to prevent longer recovery time and other surgical complications. To our knowledge this is the first case reported in the literature in which there is involvement of multiple muscle groups including the proximal region of upper and lower extremities.
